# Choroidal Thickness Profile in Chorioretinal Diseases: Beyond the Macula

**DOI:** 10.3389/fmed.2021.797428

**Published:** 2021-12-20

**Authors:** Young Ho Kim, Jaeryung Oh

**Affiliations:** Department of Ophthalmology, Korea University College of Medicine, Seoul, South Korea

**Keywords:** choroid, choroidal thickness, profile, choroidal thickness profile, choroidal profile, retinal disease, optical coherence tomography

## Abstract

Enhanced depth imaging optical coherence tomography (EDI-OCT) and swept-source OCT (SS-OCT) have emerged as essential diagnostic tools in the study and management of various chorioretinal diseases. Evidence from early clinical studies using EDI-OCT and SS-OCT indicates that choroidal dysfunction plays a major role in the pathogenesis of chorioretinal diseases. Measurement of choroidal thickness (CT) has already become a major research and clinical method, and CT is considered as an indicator of choroidal status in a variety of ophthalmic diseases. Recently, CT measurement has also been proposed as a non-invasive marker for the early detection and monitoring of various systemic diseases. Among the several possible CT measurement locations, subfoveal CT has rapidly become a reliable parameter for measuring CT in healthy and diseased eyes. Moreover, recent advancements in OCT technology have enabled faster and wider imaging of the posterior part of the eye, allowing the various changes in CT as measured outside the macula to be shown accordingly. In this review, we first provide an overview of the results of clinical studies that have analyzed the healthy macular choroid and that in various chorioretinal diseases, and then summarize the current understanding of the choroid outside the macula. We also examine the CT profile as an index that encompasses both within and outside of the macula. Furthermore, we describe the clinical applications of ultrawide OCT, which enables visualization of the far periphery, and discuss the prospects for the development of more reliable choroidal parameters that can better reflect the choroid's characteristics.

## Introduction

The choroid is a vascular coat that supports the retina to maintain stable blood flow, nutrition, and temperature (1, 2). Choroidal dysfunction contributes to the structural and functional abnormalities of the overlying retinal pigment epithelium and retina, and these relationships have been suggested to be implicated in the pathogenesis of various chorioretinal diseases (3–8). Indocyanine green angiography revealed that abnormal choroidal vascular flow was involved in the development of chorioretinal diseases such as central serous chorioretinopathy (CSC), polypoidal choroidal vasculopathy, or uveitis (4–9). With the advent of spectral domain optical coherence tomography (SD-OCT) technology, a method to measure choroidal thickness (CT) *in vivo* was introduced in ophthalmology (10, 11). Subsequently, the introduction of enhanced depth imaging (EDI) techniques and swept source optical coherence tomography (SS-OCT) have made it possible to observe topographic variations in CT (12, 13).

Variations in subfoveal CT in various chorioretinal diseases have been more extensively reported in the corresponding pathologic state than in the physiologic state (14), and these variations have been suggested to be related to the onset and progression of these chorioretinal diseases (15, 16). Recently, an association between CT variation and various systemic diseases has also been indicated (16, 17). Consequently, CT measurement has become a major research method, and CT is considered an indicator of choroidal status not only in chorioretinal diseases but also in various systemic diseases (18, 19). Subsequently, subfoveal CT has become a primary parameter to measure CT in normal and diseased eyes (14, 20–22). In addition to the macular choroid, it has been suggested that the peripapillary choroid could also be representative of the choroid of individuals (23–25). In early studies, variation in nasal peripapillary was reported in chorioretinal diseases, and measurement of the peripapillary CT showed the potential to serve as another reference point for CT measurements (26–28). Advancements in scan speeds and optics of OCT systems provided simple and easy opportunities to image a wider range of posterior segment of the eye (12, 14, 18). Several recent studies found CT variations in normal and diseased eyes both in the posterior pole and the peripheral area (29–31). In addition to CT measurement at a single representative point, the use of combinations of CT measurements at different points has been suggested as another reliable strategy for characterizing the human choroid both with and without diseases (32, 33).

In this study, we reviewed how the CT profile has progressively evolved into one of the most representative biomarkers for characterizing the human choroid in recent studies using OCT. To understand how CT measurement has become an important non-invasive indicator, we first reviewed the process of obtaining measurement reliability in healthy subjects, followed by an overview of the geographic characteristics of CT in the macula. The characteristics and variations of the CT outside the macula, particularly the peripapillary CT, were described in various chorioretinal and systemic diseases. We also presented recent clinical studies on CT measurement up to the periphery using wide-field OCT and discussed the need for and application of a novel parameter based on choroidal thickness.

## Development of Choroidal Thickness Measurement

Early studies on CT were conducted using ultrasonography (34, 35). In 1979, Coleman and Lizzi (34) estimated CT to be 420 μm at the posterior pole *in vivo* and found even thicker regions outside the macula. However, it was not easy to precisely determine the specific topographic location of the CT measurement point with ultrasonography. Advancement of OCT technology has enabled the collection of detailed topographic volume data from the posterior pole. OCT also enables imaging of the histologic structure of the fovea *in vivo*, which makes it possible to measure retinal thickness in the subfoveal and parafoveal areas.

In 2008, the introduction of EDI method using commercially available SD-OCT by Spaide et al. made it possible to reasonably visualize the deeper layer of the choroid and choroidoscleral junction (12). In early human studies using SD-OCT, reliable CT measurements could be obtained for both healthy and diseased eyes (10, 11). Branchini et al. (36) showed good reproducibility of CT measurements in healthy individuals using three different SD-OCT systems. Furthermore, SS-OCT systems using longer wavelengths added more detailed information about the outer choroidal boundary than SD-OCT (37–39). For reliable CT measurements, it is important to accurately identify the choroid-scleral junction as an outer boundary. In some studies, SS-OCT permitted accurate identification of the choroid-scleral junction in 100% of normal eyes (37, 38). Lee et al. (39) showed that SD-OCT devices had lower reliability than SS-OCT devices in eyes with CT ≥ 400 μm and subfoveal active lesions. Despite these differences, both SS-OCT and SD-OCT with the EDI technique demonstrated good reproducibility in terms of CT measurement, and both systems have become standard methods for measuring CT, especially subfoveal CT (14, 22, 39).

## Choroidal Thickness Measurement at the Macula

### From Subfovea to Perifovea

Extensive studies measuring CT focused on the choroid at the macula (14), especially the subfoveal choroid, where the overlying retina is most important in preserving visual function. We reviewed the published literature on CT using OCT in humans, with a focus on the topographic location of CT measurements. A literature search in the PubMed database (RRID:SCR_004846) in August 2021 using the terms “choroidal thickness” and “optical coherence tomography” with no restrictions identified a total of 2,734 citations published with the cut-off publication date of July 31, 2021. In Table 1, we summarize the results of the articles that specifically compare various locations of CT measurements and various chorioretinal diseases between different publication time periods. We also used combinations of relevant keywords, MeSH terms, and Boolean operators (AND and OR) to conduct literature searches. For example, in Table 1, we used the search query; subfoveal OR macular AND age related macular degeneration AND “choroidal thickness” AND optical coherence tomography to present the number of studies on subfoveal CT measurement using OCT in age-related macular degeneration.

**Table 1:** The number of citations on choroidal thickness measurements depending on the different search terms and the date of publication in PubMed database.

	**Choroidal thickness**				**Choroidal thickness + OCT**				**Choroidal thickness + OCT + subfoveal or macular**				**Choroidal thickness + OCT + peripapillary choroidal thickness**			
	**Before 2010**	**2010–2015**	**2016–2021**	**Total**	**Before 2010**	**2010–2015**	**2016–2021**	**Total**	**Before 2010**	**2010–2015**	**2016–2021**	**Total**	**Before 2010**	**2010–2015**	**2016–2021**	**Total**
Total	71	778	1,885	2,734	12	699	1,633	2,344	8	524	1,195	1,727	1	68	205	274
AMD	5	145	307	457	3	135	255	393	3	134	254	391	1	7	16	24
DR, DME	1	76	207	284	0	68	185	253	0	63	152	215	0	30	68	98
RVO	0	10	33	43	0	8	25	33	0	8	21	29	0	0	3	3
Uveitis, Ocular inflammation	5	47	118	170	2	45	109	156	2	28	79	109	1	10	31	42
PSD^*^	2	114	352	468	1	104	299	404	1	89	256	346	0	42	143	185
Glaucoma	7	91	139	237	1	78	118	197	1	45	68	114	1	32	52	85

*OCT, optical coherence tomography; AMD, age-related macular degeneration; DME, diabetic macular edema; DR, diabetic retinopathy; RVO, retinal vein occlusion; PSD, pachychoroid spectrum disease*.

* *All the following search terms were used “central serous chorioretinopathy,” “polypoidal choroidal vasculopathy,” and “pachychoroid” with Boolean operators “OR.”*

**Figure 1:**
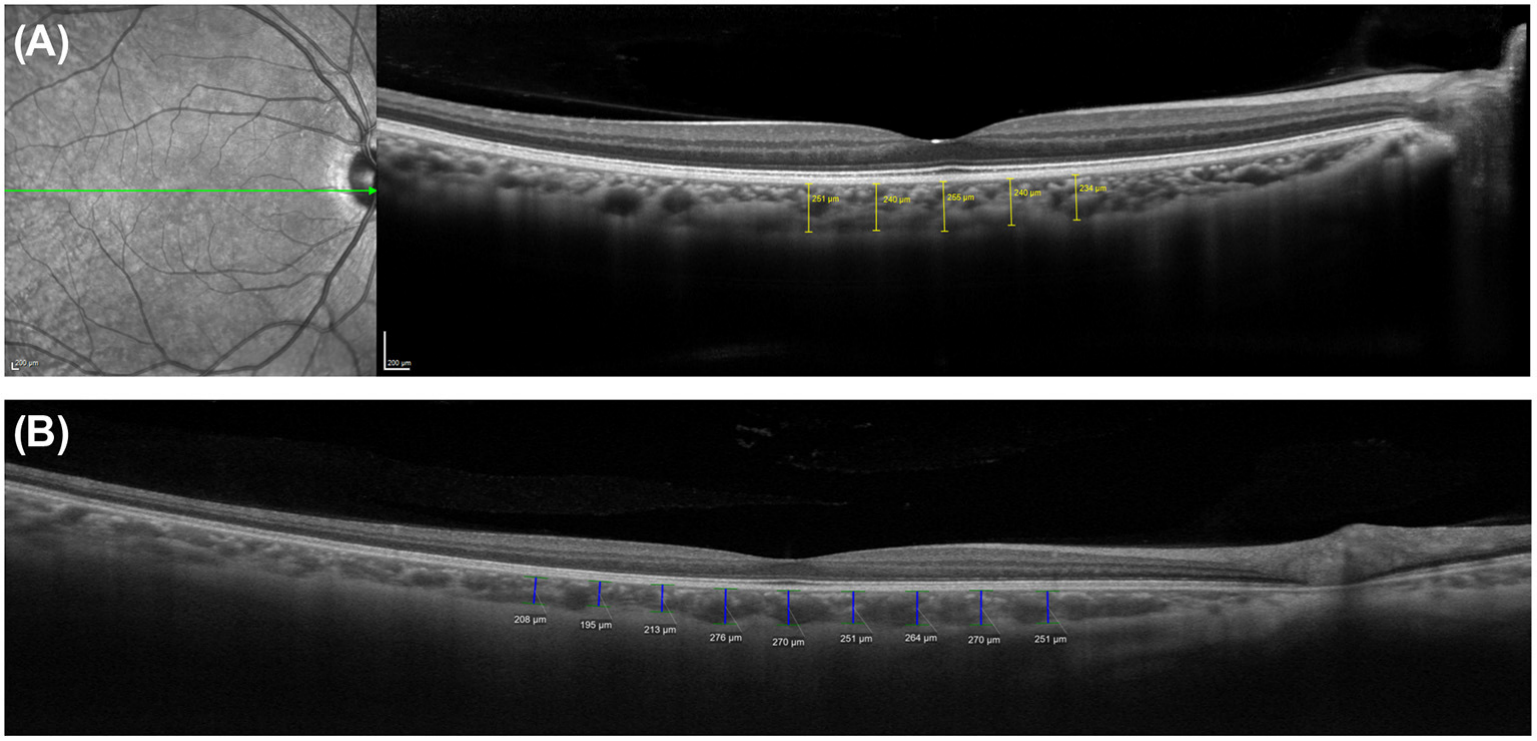
Measurements of choroidal thickness (CT) within the macula. A horizontal line scan image centered on the fovea obtained using **(A)** spectral domain optical coherence tomography with enhanced depth imaging technique (Spectralis OCT2; Heidelberg Engineering, Heidelberg, Germany) and **(B)** swept-source optical coherence tomography system (PLEX Elite 9000; Carl Zeiss Meditec, Inc, Dublin, California, USA). The choroidoscleral junction and the detail of choroid is clearly visible in both images. CT was determined by measuring the vertical distance from the retinal pigment epithelium/Bruch's membrane complex to the choroidoscleral junction. Subfoveal and macular CT was measured manually by caliper tool at the subfovea and at 500-μm intervals up to **(A)** 1,000 μm and **(B)** 2,000 μm temporal and nasal to the fovea, respectively.

To investigate geographic CT characteristics within the macular area, other locations and the subfoveal choroid on the horizontal meridian of the macula were employed for CT measurement using SD-OCT in horizontal raster scans (Figure 1). Most studies reported that within the macula, the subfoveal choroid was the thickest and decreased in thickness as the distance from the fovea increased in either direction. Manjunath et al. (10) performed high-definition raster scanning using EDI SD-OCT with frame enhancement software to measure the CT at the macula in eyes without chorioretinal disease. In this study, wherein measurements were performed at the subfovea and at 500-μm intervals from the fovea to 2,500 μm temporal and nasal to the fovea, the choroid was found to have a topographic CT pattern, with the nasal perifovea being the thinnest, the subfovea being the thickest, and the temporal perifovea being again on the thinner side. Chen et al. (40) also found that the choroid was thickest at the subfovea and thicker at the temporal perifovea than at the nasal perifovea in normal eyes. Attempts to measure CT at the macula have extended to the eyes of patients with various ocular and systemic diseases. Regatieri et al. (11) measured CT at the macula in patients with and without diabetes using the same measurement protocol and confirmed that the variation pattern of CT progressively decreased with increasing distance from the fovea and it became markedly thinner on the nasal side than on the temporal side of the fovea. In eyes without high myopia, Ikuno et al. (41) also reported that subfoveal CT was significantly greater than nasal and temporal CT using SS-OCT. However, in a study of highly myopic patients with a spherical equivalent refractive error of six diopters or more, Fujiwara et al. (20) found that the temporal perifoveal choroid was thickest, and that CT progressively decreased underneath the fovea and nasal perifoveal choroid along a 6-mm horizontal scan line. Ho et al. (42) also reported comparable results in terms of CT variations in myopic eyes. These results highlight that even in the absence of retinal disease, variations in CT may exist secondary to chorioscleral structural changes such as axial elongation of the eyeball and stretching of the choroid in myopic eyes. Therefore, the refractive error or axial length must be considered in studies of choroidal variations.

### Measurement at Superior and Inferior Perifovea

In addition to the horizontal meridian based on the foveal center, various attempts have been made to widen the measurement in other directions at the macula. Several studies have measured CT along the vertical meridian superior and inferior to the fovea (40, 41). In a study using SS-OCT, Ikuno et al. (41) reported that the superior choroid was significantly thicker than the inferior choroid. Using EDI SD-OCT choroidal imaging, Chen et al. (40) also reported significant topographic variations in CT within the macular region. They found that the choroid was thickest at the subfovea, followed by that in the superior perifovea, and then the choroid was least thick in the in inferior perifovea. They also pointed out that there is no consensus on what a “normal” CT profile of the macula is in the current literature. As knowledge of the regional variations in CT accumulate, it is possible to improve the understanding of the choroidal profile of the macula in healthy subjects and its variations in patients with various diseases.

### Three-Dimensional Thickness Map

**Figure 2:**
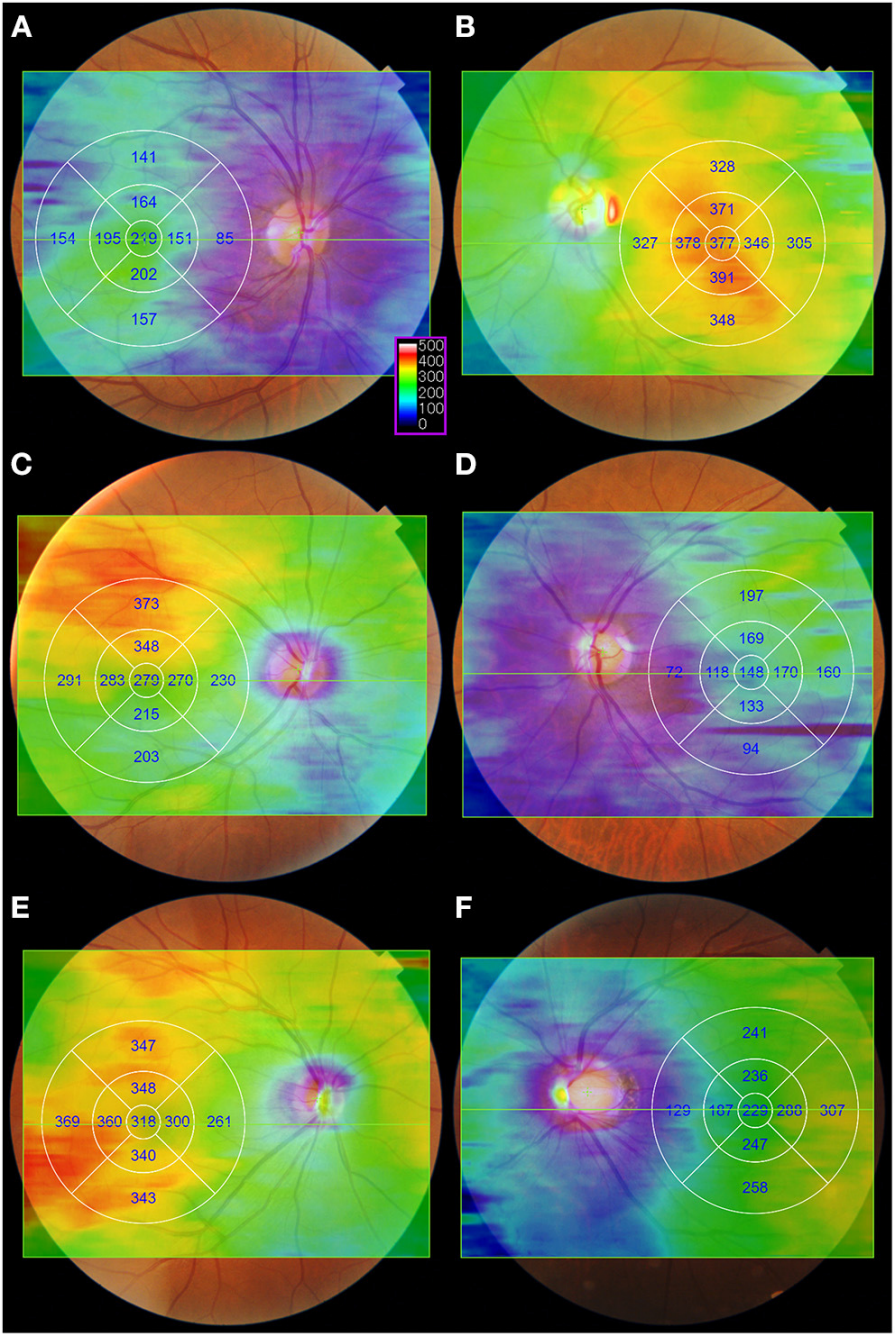
Examples of the color-coded choroidal thickness (CT) map in healthy eyes. The CT maps were generated automatically from a 12 × 9 mm three-dimensional volume scan consisting of a 256 B-scan with 512 A-scans per B-scan. Images were obtained using swept-source optical coherence tomography system (DRI OCT Triton, software version 10.17.003.01; Topcon Corp., Tokyo, Japan). A color-coded CT map and early treatment diabetic retinopathy study (ETDRS) grid were superimposed on the fundus photography using the built-in software. After the automatic layer segmentation, a color-coded CT map was obtained with the Bruch's membrane and choroidoscleral interface selected as the inner and outer limits of segmentation lines, respectively. The color scale (purple box) shows the choroidal thickness in microns. On a color-coded CT map, the warmer colors represent the thicker areas, while the cooler colors represent the thinner regions. **(A,B)** Representative cases of eyes with the greatest CT at the fovea. **(C,D)** Representative cases of eyes with the greatest CT it the superotemporal region. **(E)** Representative case of eyes with the greatest CT it the inferotemporal region. **(F)** Representative cases of highly myopic eyes with the greatest CT in the temporal region.

Since EDI-OCT method requires the averaging of many B-scans to acquire good choroidal images, fewer averaged raster scans can be obtained using EDI-OCT than by using SS-OCT (12, 43). Hirata et al. (43) indicate that CT measurements through only a few scans are more susceptible to ill-defined inner choroid-scleral borders, as well as focal thickening or thinning of the choroid. Using SS-OCT, they produced a CT map of the macular area using a three-dimensional (3D) volumetric scan protocol in healthy eyes and applied an early treatment diabetic retinopathy study (ETDRS) grid (Figure 2). They showed that CT in the nasal quadrant was lower than that in the other quadrants. In addition, the choroid was thicker in the outer superior field than in the outer inferior field of the ETDRS grid. They also showed that the subfoveal CT was similar to that in the superior temporal quadrant, however, it was greater than that in the inferior nasal quadrant. Heirani et al. (44) demonstrated that the choroid was thickest at 1 mm superior to the fovea and thinnest at 3 mm nasal to the fovea. Recently, using SS-OCT system with a 12 × 12-mm 3D volume scan centered on the fovea, Breher et al. (45) showed that CT gradually decreased with an increasing distance from the fovea. A topographic pattern with thicker choroids was observed in the order of upper, temporal, lower, and nasal side of the choroid. These studies generally found that the superior and temporal choroid was thicker than the inferior and nasal choroid, but they also revealed that choroidal geography varied greatly among healthy subjects.

Furthermore, not all OCT systems provide a volumetric CT map with a built-in software, and it is difficult to obtain a reliable CT map automatically in diseased eyes due to segmentation errors. The newer image processing techniques, such as variety automatic segmentation and edge detection algorithms, along with the increased application of artificial intelligence, have allowed ophthalmologists to access a vast amount of 3D volumetric OCT data, which would otherwise be time-consuming for manual analysis (46, 47). These advances have enabled more accurate and reliable CT measurements over a wider range, rather than just one part of the macula.

### Limitation of Measurement at Macula

The normal range of subfoveal CT has still not been definitively established (14, 48). Additionally, many factors, including age (20, 41, 43, 44, 49), refractive error (20, 41, 44, 49), and axial length (20, 40, 43, 44), have been postulated to affect subfoveal CT, and the physiologic diurnal variations (50–56) in CT have also been described. Moreover, several studies observe that CT changes even after light exposure and daily activities such as physical exertion, drinking water, and smoking (13, 56–58). As a result, evaluating a patient based only on the basis of the absolute value of CT measured at one site and one time point has limitations, and confounding factors such as age, axial length, refractive error, and diurnal variation must be considered. To compensate for the limitations of the subfoveal CT, an increase in the number of ophthalmology studies using CT measurements has led to the measurement of CT not only at the subfovea, but also at various locations in the posterior part of the eye.

## Choroidal Thickness Measurement Beyond the Macula

**Figure 3:**
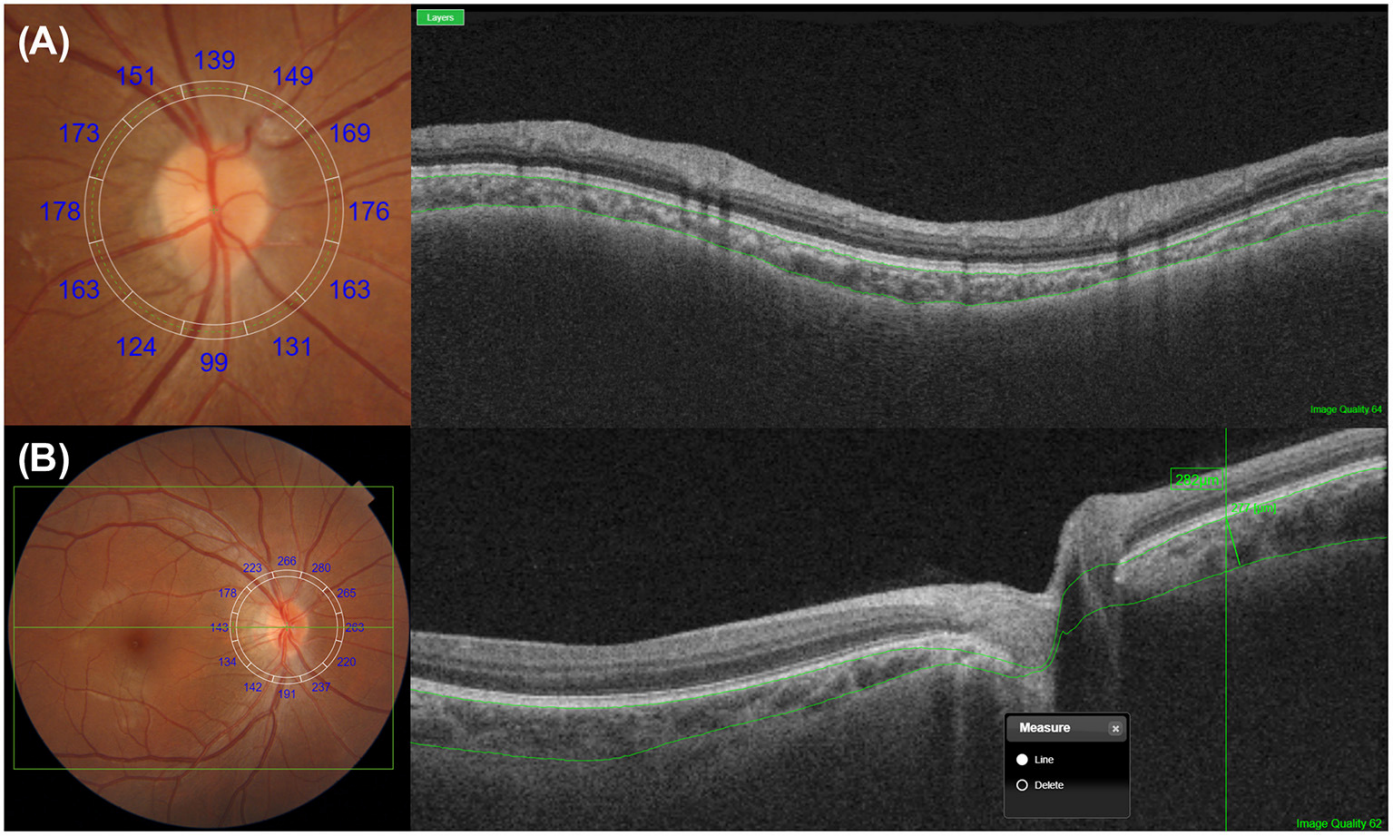
Representative methods for the measurements of peripapillary choroidal thickness (CT). **(A)** Peripapillary CT can be measured over 360° along a 3.4-mm circular grid, which is used for retinal nerve fiber layer analysis. Depending on the devices, the upper and lower segmentation lines are either automatically or manually selected as the Bruch's membrane and the choroidoscleral junction, respectively. After segmentation, peripapillary CT is determined automatically using built-in manufacturer's software, either by the sectorial or overall average. **(B)** Peripapillary CT can be measured manually at a point of interest with a similar method for macular CT measurement. A circular RNFL grid centered on the optic disc can be used to improve the consistency of the measurement position.

CT measurement is not limited to the macular areas (42, 59–62). When literature searches were conducted in August 2021 using the PubMed database (RRID:SCR_004846) with no restrictions and the search terms “subfoveal choroidal thickness” and “OCT,” a total of 1,727 citations were identified accordingly. When the terms “peripapillary choroidal thickness” and “OCT” were used, a total of 274 citations were identified further. Spaide (63) was the first to measure temporal peripapillary CT in eyes with age-related choroidal atrophy in his early study. Oh et al. (64) proposed a method to measure peripapillary CT using retinal nerve fiber layer scanning in patients with glaucoma (Figure 3). It is known that both age and myopia are correlated with variations in peripapillary CT (63–67), and many studies have been conducted to analyze these variations in normal subjects and glaucoma patients (62, 68). Most of these studies focus on determining the relationship between peripapillary CT and optic nerve damage, such as glaucoma and optic neuropathy (68–71).

### Peripapillary Choroidal Thickness

#### At Chorioretinal Diseases

**Figure 4:**
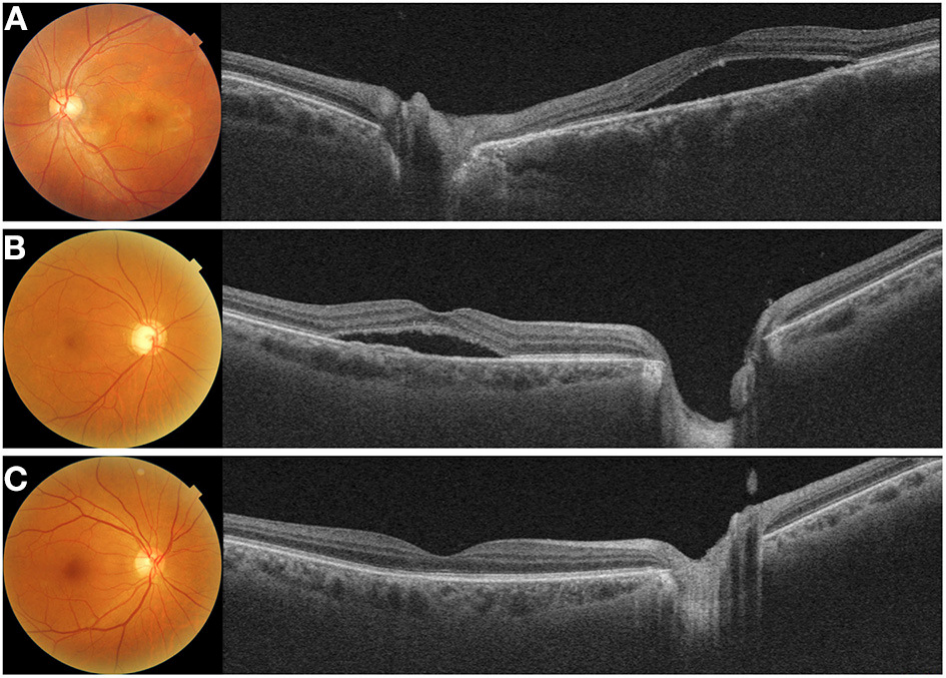
Representative cases with **(A)** acute central chorioretinopathy (CSC), **(B)** chronic CSC, and **(C)** resolved CSC. **(A)** A 38-year-old male with acute CSC showed diffuse thickening of both the macular and peripapillary choroid with dilated large choroidal vessels on a cross-sectional swept-source optical coherence tomography (SS-OCT) image. **(B)** A 69-year-old male with chronic CSC had increased subfoveal choroidal thickness (CT) and showed shallow irregular pigment epithelial detachment on and SS-OCT image. Peripapillary choroid was also thickened with dilated pachyvessels. **(C)** A 69-year-old male with resolved CSC showed diffuse thickening of the macular choroid. But, this patient had a relatively thinner peripapillary choroid.

Several investigators have investigated peripapillary CT in various diseases and have attempted to determine its significance in the development of chorioretinal diseases (Table 2) (26, 27, 32, 33, 62, 65, 66, 72–82). The choroid covers the posterior two-thirds of the eye, whereas the subfoveal choroid is only one part of the choroid. Many chorioretinal diseases can either affect regions outside the macula or they may affect both regions within the macula and those outside of it (73, 83–86). Peripapillary CT measurement has been proposed as one of the useful methods for assessing CT outside of the macula. Yun et al. (26) raised the question of whether measuring only subfoveal or parafoveal CT could be representative of the choroid as a whole. CSC is well-known to be associated with subfoveal choroidal thickening on OCT (Figure 4) (15, 87). They measured nasal peripapillary CT outside the macula, as well as subfoveal CT in patients with CSC. They found that both nasal peripapillary and subfoveal CT were thicker in the affected eyes and fellow eyes of the CSC patient group than in the normal controls.

**Table 2:** Findings of peripapillary choroidal thickness measurement in chorioretinal diseases.

**References**	**Subfoveal CT**	**Parafoveal CT**		**Peripapillary CT**		**Diseases**	**Key findings**
		**Within 1.5 mm**	**Within 5.5 mm**	**Within macula**	**Outside macula**		
Yun et al. (26)	O	–	–	O	O	CSC and healthy subjects	Nasal peripapillary CT in both CSC eyes and fellow eyes was higher than in normal eyes.
Yun et al. (27)	O	O	–	O	O	Early AMD	Peripapillary CT outside the macula was lower in eyes with reticular pseudodrusen than in those without reticular pseudodrusen.
Nam et al. (72)	O	O	O	O	O	Early AMD	Peripapillary CT in patients with non-exudative age-related macular degeneration had variations depending on the type of drusen.
Kim et al. (32)	O	–	–	–	O	Healthy subjects, early AMD, wet AMD, pachydrusen, PNV, and PCV	A cluster analysis based on CT profiles, including subfoveal CT, peripapillary CT, and their ratio, revealed that the clustering pattern differed between eyes with AMD, and pachychoroid spectrum diseases.
Phasukkijwatana et al. (73)	O	O	O	O	–	Peripapillary pachychoroid syndrome, CSC, PNV, and healthy subjects	Peripapillary CT is associated with nasal macular fluid in peripapillary pachychoroid syndrome.
Baek et al. (74)	O	O	O	O	–	PCV	Subfoveal CT in peripapillary PCV is significantly lower than in macular PCV.
Kim et al. (75)	O	–	–	–	O	exudative AMD, PNV, and healthy subjects	The ratio of subfoveal CT to nasal peripapillary CT was consistent with aging. The ratio is different between pachychoroid neovasculopathy or classic exudative AMD.
Kim et al. (33)	O	–	–	O	O	PCV	The ratio of subfoveal to nasal CT showed the best predictor of early response in treatment of PCV
Kim et al. (76)	O	–	–	–	O	CSC and healthy subjects	Peripapillary CT was increased in patients with acute and chronic CSC, but not in patients with resolved CSC.
Kang et al. (77)	O	–	–	O	O	BRVO	In patients with unilateral BRVO, peripapillary CT decreased significantly in eyes with BRVO and fellow eyes over a 12-month period.
Lee et al. (78)	O	–	–	O	O	BRVO	Peripapillary CT in BRVO patients decreased in all sectors of the RNFL grid over a 6-month period.
Sirakaya and Kucuk (79)	–	–	–	O	O	BRVO and healthy subjects	In patients with unilateral BRVO, the average, superior, and inferior peripapillary CT in both eyes were significantly lower, and nasal peripapillary CT in affected eyes was lower than in control eyes.
Kang et al. (80)	O	–	–	O	O	BRVO, normal-tension glaucoma, and vitreous floater	The mean peripapillary CT in patients with BRVO and normal-tension glaucoma was significantly decreased in eyes with focal laminar cribrosa defect than in those without it.
Cetin et al. (81)	O	–	–	O	O	Retinitis pigmentosa	Peripapillary CT was thinner in eyes with disorganization of the retinal inner layers, whereas macular CT did not change significantly.
Balci and Turan-Vural (82)	–	–	–	O	O	Ocular sarcoidosis and healthy subjects	Peripapillary CT was significantly thinner in patients with both ocular sarcoidosis and glaucoma than in healthy controls.

*CT, choroidal thickness; CSC, central serous chorioretinopathy; AMD, age-related macular degeneration; PNV, pachychoroid neovasculopathy; PCV, polypoidal choroidal vasculopathy; BRVO, branch retinal vein occlusion; RNFL, retinal nerve fiber layer*.

**Figure 5:**
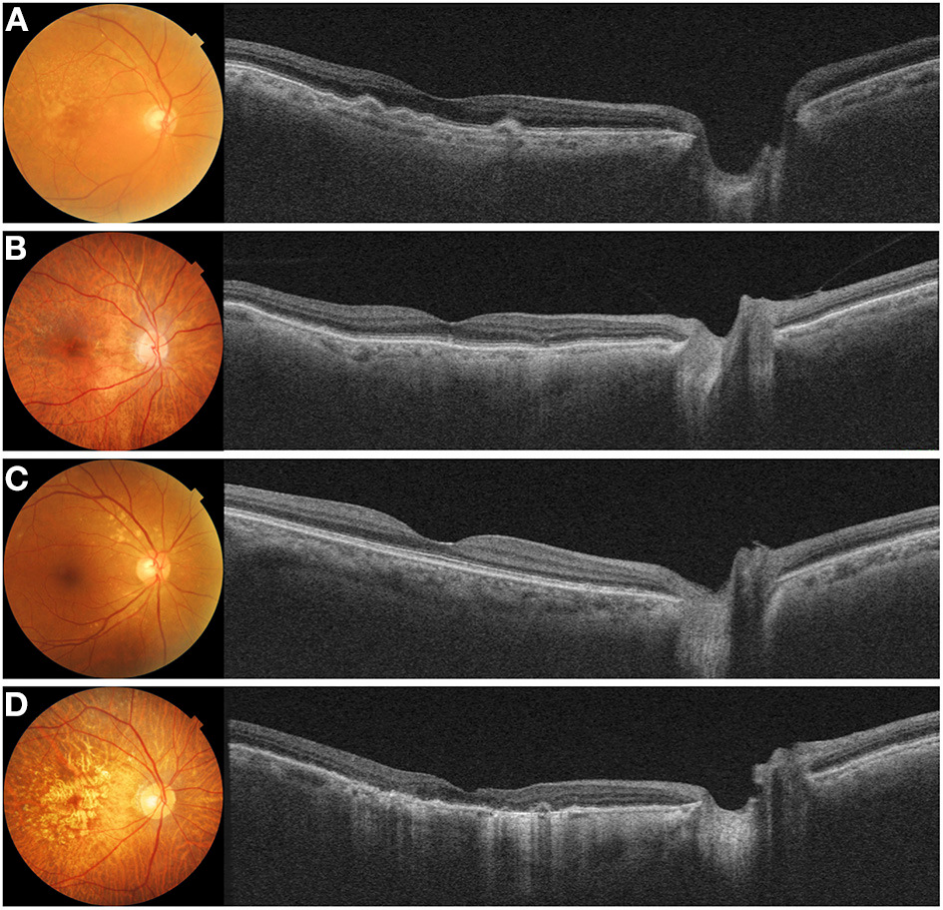
Color fundus photograph and swept-source optical coherence tomography (SS-OCT) image in eyes with **(A)** soft drusen, **(B)** reticular pseudodrusen, **(C)** pachydrusen, and **(D)** geographic atrophy. **(A)** Fundus photograph of an 85-year-old male showed multiple soft drusen in the macular. SS-OCT image showed several dome-shaped retinal pigment epithelium (RPE) elevations with medium internal reflectivity and diffuse choroidal thinning in the macula and peripapillary area. **(B)** Fundus photograph of an 83-year-old female showed numerous reticular pseudodrusen in the macula. A cross-sectional SS-OCT image demonstrated multiple hyperreflective material lies above the RPE. Both the macular and peripapillary choroid demonstrated generalized thinning. **(C)** Fundus photograph of a 65-year-old female showed pachydrusen with various sizes scattered along the vascular arcade. The choroid in the macular and peripapillary regions was not thinned significantly on SS-OCT image. **(D)** Fundus photograph of an 85-year-old female demonstrated multiple confluent well-demarcated atrophic lesions in the macula. A cross-sectional SS-OCT image showed thinning of the outer retina and hypertransmission. A marked thinning of the macula and peripapillary choroid is observed.

A trial to measure CT outside the macula has been extended to age-related macular degeneration (Figure 5). Switzer et al. (22) found that eyes with reduced subfoveal CT were more likely to have reticular pseudodrusen. Yun et al. (27) measured the CT outside the macular area and showed that peripapillary CT outside the macula was lower in eyes with reticular pseudodrusen than in those eyes without reticular pseudodrusen, in addition to that in the macula (Figure 5B). These findings were confirmed by Nam et al. (72), who reported that peripapillary CT in patients with non-exudative age-related macular degeneration showed variations according to the accompanying drusen type. Kim et al. (32) also showed that the peripapillary CT of eyes with pachydrusen was higher than that of eyes with soft drusen (Figure 5C). Kim et al. (75) also showed that nasal peripapillary CT outside the macula was different between pachychoroid neovasculopathy and classic exudative age-related macular degeneration. These studies found similar trends in the alteration of the peripapillary and subfoveal CTs in each chorioretinal disease. These findings suggest that peripapillary CT can be useful in disorders where measuring subfoveal CT is difficult, such as in patients with CSC or exudative AMD.

In addition, several studies have reported changes in peripapillary CT that differ from subfoveal CT. Phasukkijwatana et al. (73) reported that peripapillary choroidal syndrome is caused by a disproportionate thickening of the peripapillary choroid relative to the macular choroid. Recently, Kim et al. (76) reported that peripapillary CT increased in patients with acute and chronic CSC; however, this was not observed in patients with resolved CSC (Figure 4). As further research proceeds, it is expected that peripapillary CT will be more helpful.

**Figure 6:**
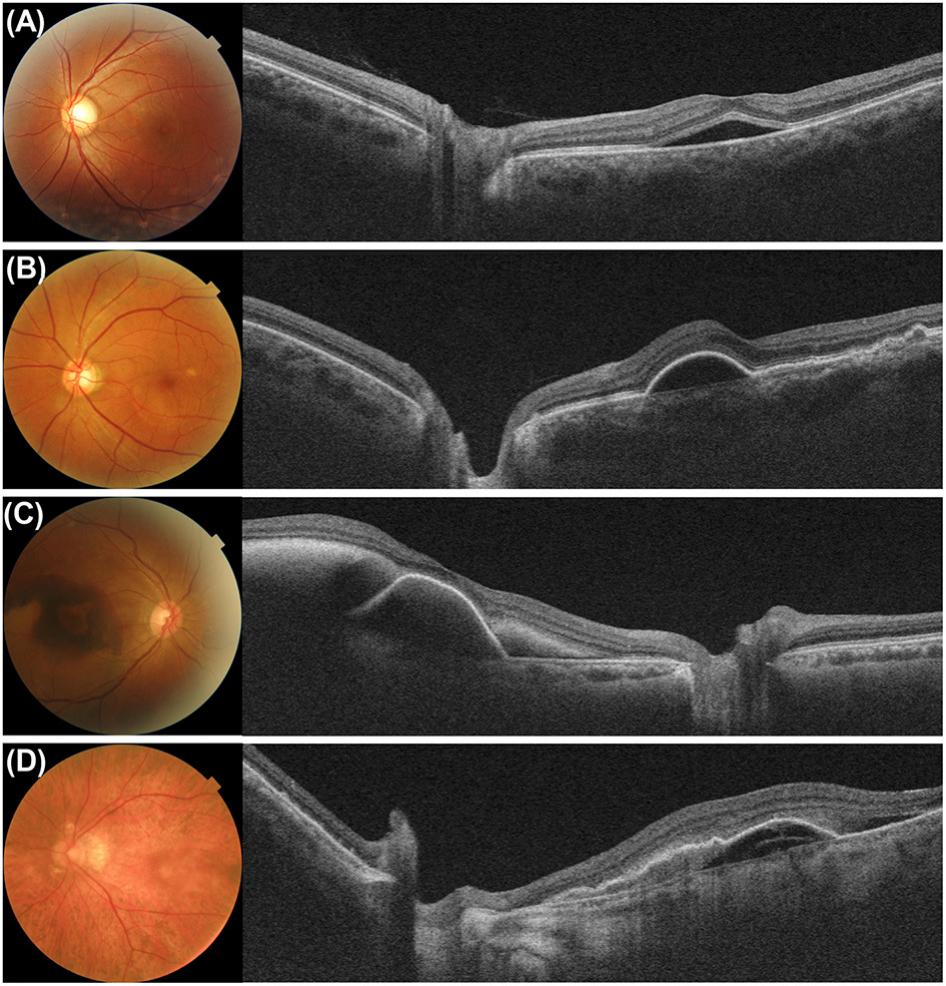
Examples of eyes with active macular disease of **(A)** central chorioretinopathy, **(B)** pachychoroid neovasculopathy, **(C)** polypoidal choroidal neovasculopathy, and **(D)** exudative age-related macular degeneration. Because the outer boundary of the subfoveal choroid is not readily visible in cases of active diseases such as accumulation of subretinal fluid, blood, and pigment epithelial detachment in the macula, measuring the subfoveal choroidal thickness are not always possible. Even in these cases, the peripapillary choroidal thickness can be measured.

The usefulness of peripapillary CT measurements extends to retinal vascular diseases. Kang et al. (77) demonstrated that peripapillary CT decreased significantly over 12 months in eyes with branch retinal vein occlusion (BRVO) and in fellow eyes in patients with unilateral BRVO. Lee et al. (78) also analyzed the peripapillary CT in eight sectors and showed that it significantly decreased in all sectors over 6 months in eyes with BRVO. Sirakaya and Kucuk (79) showed that the average, superior, and inferior peripapillary CT in both eyes of patients with unilateral BRVO were significantly lower and nasal peripapillary CT in the affected eyes was lower than that in the control eyes. Kang et al. (80) found that the mean peripapillary CT in patients with BRVO and normal-tension glaucoma was significantly lower in eyes with focal laminar cribrosa defects than in those without it. In a recent study, Cetin et al. (81) extended the measurement of peripapillary CT in patients with retinitis pigmentosa. Balci and Turan-Vural measured peripapillary CT in eyes with ocular sarcoidosis and glaucoma (82). These studies highlighted the application of CT measurements outside the macula in a variety of retinal disorders. Although the pathophysiological and clinical significance of these CT regional variations observed outside the macula has not yet been established, it is suggested that analyzing a wider field of view could be useful for investigating CT abnormalities in diseased eyes (Figure 6).

#### At Systemic Diseases

The choroid is composed mostly of a dense network of blood vessels with the highest blood flow per unit weight in the human body (2). This highly vascularized nature of the choroid, which can be assessed by non-invasive imaging, has drawn attention to the choroid in a variety of systemic diseases, such as the metabolic, inflammatory, and the systemic vascular disease (88). Peripapillary CT measurements have also been attempted in patients with systemic illnesses (24, 89–93) such as diabetes (23, 94–97), thyroid diseases (98, 99), and pulmonary diseases (25, 100) (Table 3). Yazgan et al. (94) showed that the macular and peripapillary choroid was significantly thicker in pre-diabetic patients than in healthy controls at all 15 measuring points. Vujosevic et al. (95) showed that both macular and peripapillary CT decreased with advancement of the diabetic retinopathy stage. Peripapillary CT was lower in type 2 diabetic patients with impaired renal function in a study by Liu et al. (23), and it was positively correlated with the estimated glomerular filtration rate. Loureiro et al. (89) reported that peripapillary CT in the temporal and inferotemporal sectors was significantly lower in patients with metabolic syndrome than in normal controls. Changes in CT not only in the macula but also outside the macula support the hypothesis that choroidopathy occurs in patients with systemic vascular diseases.

**Table 3:** Findings of peripapillary choroidal thickness measurement in systemic disease or after medication administration.

**References**	**Subfoveal CT**	**Parafoveal CT**		**Peripapillary CT**		**Diseases**	**Key findings**
		**Within 1.5 mm**	**Within 5.5 mm**	**Within macula**	**Outside macula**		
Yazgan et al. (94)	O	O	O	O	O	Pre-diabetes, healthy subjects	Macular and peripapillary choroid was significantly thicker in pre-diabetes than in normal controls.
Vujosevic et al. (95)	O	O	O	O	O	DM, healthy subjects	Both macular and peripapillary CT decreased as the stage of diabetic retinopathy progressed.
Liu et al. (23)	–	–	–	O	O	Type 2 DM with/without CKD	Peripapillary CT was significantly lower in diabetes with CKD than in diabetes without CKD, and it had a positive correlation with the estimated glomerular filtration rate.
Loureiro et al. (89)	–	–	–	O	O	Metabolic syndrome, healthy subjects	Peripapillary CT in temporal and inferotemporal sectors was significantly lower in patients with metabolic syndrome than in healthy subjects.
Chang et al. (24)	O	O	O	–	O	Hemodialysis patients	Subfoveal and peripapillary CT outside the macula decreased after hemodialysis.
Lee et al. (90)	O	–	–	O	O	End-stage kidney disease	Peripapillary CT in all sectors, as well as macular CT, decreased after hemodialysis.
Turan-Vural and Vural (91)	O	–	–	O	–	Patients with a history of coronary artery disease with/without carotid artery stenosis	Subfoveal and peripapillary CT decreased as the degree of carotid artery stenosis increased.
Tsapardoni et al. (92)	O	O	–	O	O	Transfusion-dependent beta-thalassemia, healthy subjects	Subfoveal and peripapillary choroids were thinner in the beta-thalassemia compared to controls.
Gul et al. (98)	O	O	O	O	O	Thyroid eye disease	Subfoveal and peripapillary choroid was thicker in patients with active thyroid eye disease than in stable patients, although the peripapillary CT showed no significant differences.
Lai et al. (99)	O	O	O	O	O	Thyroid eye disease, healthy controls	The choroid within the macula was thicker in patients with thyroid eye disease than in controls. But, peripapillary CT outside the macula did not differ from controls.
Ozcimen et al. (25)	O	–	–	O	O	COPD, healthy subjects	Subfoveal and peripapillary CT in patients with COPD tended to be lower than those in healthy control group, but the difference was not significant.
Yazgan et al. (100)	O	–	–	O	O	Sleep apnea-hypopnea syndrome, healthy subjects	Subfoveal CT was lower in all sleep apnea-hypopnea syndrome subgroups, but peripapillary CT in all sectors were lower only in the moderate and severe subgroups than in healthy subjects.
Andrade et al. (101)	O	O	O	O	O	Huntington's disease, healthy subjects	Macular CT were significantly reduced in Huntington's disease, but peripapillary CT was not
Afonso et al. (102)	O	–	–	O	O	Idiopathic normal pressure hydrocephalus, healthy subjects	Patients with non-shunted idiopathic normal pressure hydrocephalus had significantly reduced subfoveal and peripapillary CT than healthy controls and shunted patients.
Yazgan et al. (103)	O	O	–	O	O	Acromegaly, healthy subjects	Patients with acromegaly had significantly higher macular and peripapillary CT than healthy controls.
Garcia-Martin et al. (104)	–	–	–	O	O	Parkinson's disease, healthy subjects	Peripapillary CT was thicker in Parkinson's disease compared with healthy controls.
Satue et al. (105)	O	O	O	O	O	Parkinson's disease, healthy subjects	Both macular and peripapillary CT were higher in Parkinson's disease patients compared to those in healthy subjects.
Garcia-Martin et al. (106)	–	–	–	O	O	Multiple sclerosis, healthy subjects	Peripapillary CT was lower in multiple sclerosis patients than in age- and sex-matched controls
Macias et al. (107)	–	–	–	O	O	Spaceflight-associated neuro-ocular syndrome	Peripapillary choroid thickening, along with optic disc edema, developed gradually during the spaceflight and persisted for up to 30 days after the mission.
Fieß et al. (108)	–	–	–	O	O	Children (4–10 years) born prematurely (≤32 weeks) and full term (≥37 weeks)	Prematurity itself does not affect peripapillary CT.
Li et al. (96)	O	O	O	O	O	Children (<16 years) with type 1 DM without retinopathy, healthy subjects	Inferior and nasal parapapillary CT were increased in children with type 1 DM and without retinopathy or visual impairment compared to that in healthy controls
Ermerak et al. (97)	O	O	–	O	O	Children (7–18 years) with type 1 DM without retinopathy, healthy subjects	Nasal and inferior peripapillary CT were lower in children with type 1 DM who have no retinopathy than in healthy controls, although macular CT did not differ.
Yavuz and Ozcimen (109)	O	–	–	O	O	Severe acne vulgaris patients receiving systemic isotretinoin treatment	The changes in peripapillary CT in the superotemporal and temporal quadrant were significant after systemic isotretinoin treatment.
Casado et al. (110)	O	O	O	–	O^*^	Healthy controls receiving topical phenylephrine	Macular CT was reduced at several measuring locations, as well as peripapillary CT at all two points was decreased after topical phenylephrine instillation.
Vural et al. (93)	O	–	–	O	O	Vitamin D deficiency, healthy subjects	Patients with vitamin D insufficiency had lower subfoveal CT as well as inferior and nasal peripapillary CT.

*CKD, chronic kidney disease; COPD; Chronic obstructive pulmonary disease; CT, choroidal thickness; DM, diabetes mellitus*.

* *The superior and inferior sectors of the peripapillary choroidal thickness were measured, but not the nasal sector*.

Correlations between hemodynamic changes and CT variations have also been suggested in literature. Chang et al. (24) showed that peripapillary CT in all sectors, as well as macular CT, decreased after hemodialysis. Lee et al. (90) also showed a decrease in macular and peripapillary CT shortly after hemodialysis, however, the decrease was not significantly correlated with changes in mean ocular perfusion pressure. Turan-Vural et al. (91) studied the relationship between CT variation and ocular perfusion in patients with carotid artery stenosis. Their results showed that thinner subfoveal and peripapillary CT were associated with worsening of the degree of carotid artery stenosis. Tsapardoni et al. (92) studied choroidal variation in patients with transfusion-dependent beta-thalassemia and reported that subfoveal and peripapillary CT were lower in patients with beta-thalassemia than in healthy controls. They suggested that chronic anemia and underlying choroidal hemodynamic changes possibly result in choroidal thinning in patients with beta-thalassemia. These studies thus suggested that subfoveal and peripapillary CT measurements could indirectly and non-invasively reflect ocular and systemic perfusion status.

The association between thyroid eye diseases and changes in the choroid has been studied with respect to autoimmune inflammatory disease. Gul et al. (98) measured macular and peripapillary CT in patients with thyroid eye diseases associated with Graves' disease, nodular goiter, and Hashimoto's thyroiditis and found that the subfoveal choroid was thicker in patients with active thyroid eye disease than in those with a stable state. Peripapillary CT tended to be higher in the active group, however, the difference was not statistically significant. Lai et al. (99) reported that the choroid at the subfovea and at the macular area within 2 mm from the foveal center was thicker in patients with thyroid-associated orbitopathy than in controls, however, the peripapillary choroid did not differ from that in normal controls. Choroid thickness has been proposed as an inflammatory marker for a variety of systemic inflammatory diseases, but little is known about its clinical utility.

Regarding peripapillary CT in patients with pulmonary diseases, Ozcimen et al. (25) showed that subfoveal and peripapillary CT in patients with chronic obstructive pulmonary disease tended to be lower than those in the healthy control group, however, the difference was not statistically significant. Yazgan et al. (100) measured subfoveal and peripapillary CT in patients with sleep apnea-hypopnea syndrome and normal controls and found that subfoveal CT was lower in all sleep apnea-hypopnea syndrome subgroups than in the control group. However, peripapillary CT in all sectors was lower only in the moderate and severe subgroups than in the healthy controls.

#### At Neurodegenerative Diseases

Anatomically and developmentally, the retina is known as an extension of the CNS (111); therefore, studies using OCT have been actively conducted in many neurodegenerative diseases (19). This close relationship highlights the optic nerve, and meaningful quantitative alterations in retinal thickness and volume have been demonstrated in patients with cognitive impairment and neurodegenerative diseases (19, 112). In addition, a growing number of studies have analyzed peripapillary CT in neurologic diseases (101–106). Andrade et al. (101) demonstrated that macular CT was significantly reduced in patients with Huntington's disease compared to that in controls. However, no differences were observed in the peripapillary choroidal measurements. Afonso et al. (102) showed that patients with non-shunted idiopathic normal pressure hydrocephalus had significantly reduced subfoveal and peripapillary CT than healthy controls and shunted patients. However, the subfoveal and peripapillary CT did not differ significantly between shunted patients and healthy controls. Yazgan et al. (103) reported that patients with acromegaly had significantly higher macular and peripapillary CT than healthy controls. Garcia-Martin et al. (104) reported that the peripapillary choroid was significantly thicker in patients with Parkinson's disease than in age- and sex-matched controls. Satue et al. (105) also showed that both macular and peripapillary CT were higher in patients with Parkinson's disease than in healthy controls. Garcia-Martin et al. (106) reported that peripapillary CT was lower in patients with multiple sclerosis than in age- and sex-matched controls. Macias et al. (107) studied the choroidal changes in astronauts with space-flight-associated neuro-ocular syndrome and found that peripapillary choroid thickening, along with disc edema, developed gradually during spaceflight and persisted for up to 30 days after the mission. Although CT within and outside the macula has been studied in various neurodegenerative diseases, further studies are needed before CT can be used as a marker for the early detection and progression of these diseases.

#### At Other Conditions

Children have also been subjected to peripapillary CT measurements. Fieß et al. (108) reported that premature birth itself does not affect peripapillary CT, however they found that infants born small for their gestational age had peripapillary choroidal thinning when compared to those infants born at the appropriate gestational age. Li et al. (96) measured macular and parapapillary CT in children with diabetes and reported that inferior and nasal parapapillary CT were increased in children with type 1 diabetes and without retinopathy or visual impairment as compared to healthy controls. However, Ermerak et al. (97) also showed that nasal and inferior peripapillary CT were lower in children with type 1 diabetes who had no retinopathy than in normal controls, although macular CT did not differ between the groups.

In addition to systemic diseases, peripapillary CT measurements have also been used in studies on the effects of drugs or nutritional deficiencies. Yavuz and Ozcimen (109) reported that there were significant changes in peripapillary CT in the superotemporal and temporal quadrants after systemic isotretinoin treatment for severe acne vulgaris. Casado et al. (110) investigated the effect of topical phenylephrine instillation on macular and peripapillary CT. Peripapillary CT was significantly reduced after phenylephrine instillation. In a horizontal macular OCT scan, CT was reduced at the subfovea and at 500, 1,000, and 1,500 μm temporal to the fovea, but not inferior to the fovea. CT at the subfovea and only the area within 500 μm from the fovea was reduced in a vertical scan. Vural et al. (93) reported that patients with vitamin D deficiency had lower subfoveal CT, as well as inferior and nasal peripapillary CT.

These findings in current studies of ocular and systemic diseases indicate that both macular and peripapillary CT have been employed to account for variations in CT. This could suggest that researchers are expanding the number of landmarks used for CT measurements.

### Choroidal Thickness Measurement to Far Periphery

**Figure 7:**
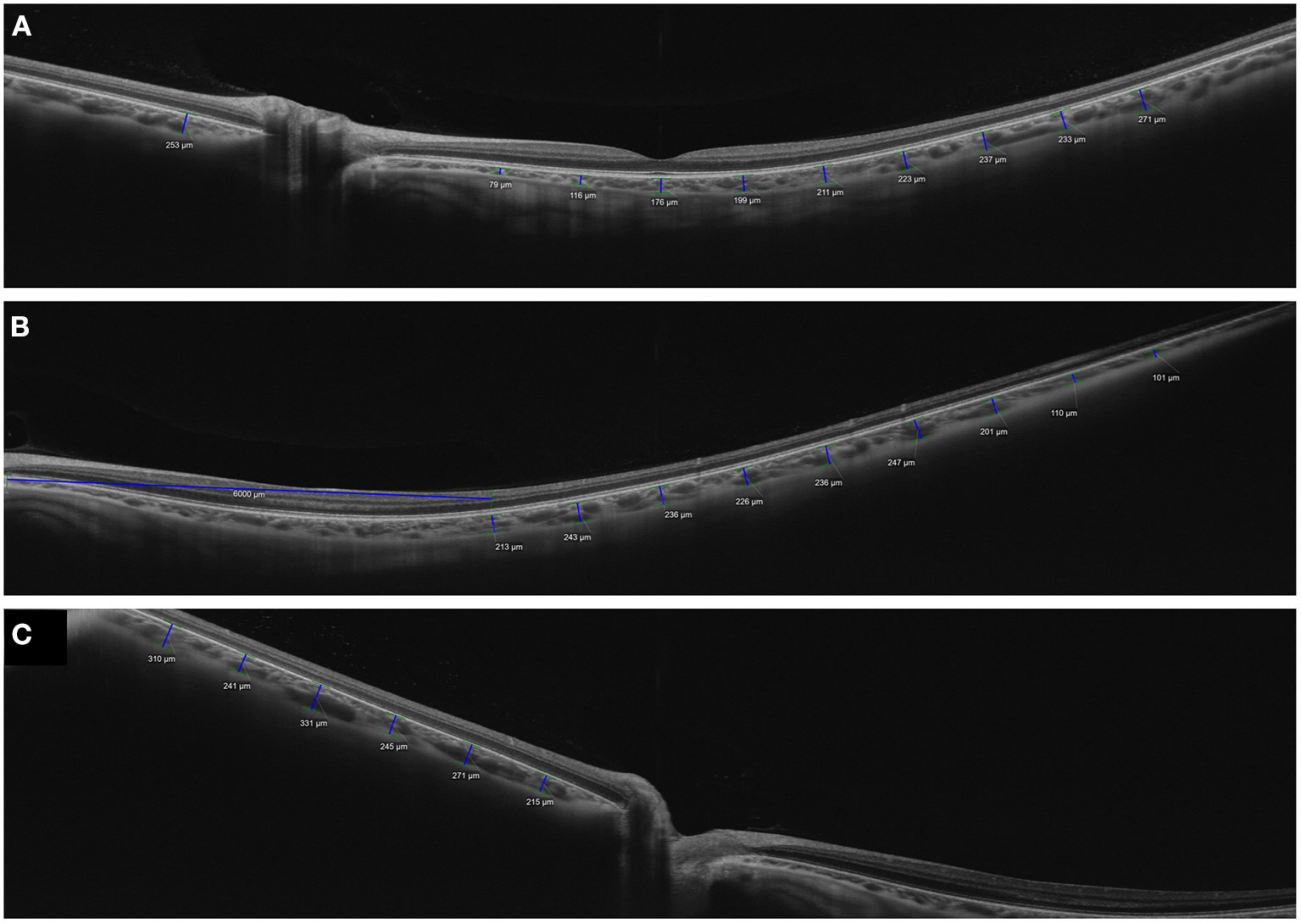
Measurements of choroidal thickness in the macular and peripheral areas using wide-field optical coherence tomography. A wide-field 16-mm high-definition horizontal line scans image of a healthy volunteer was acquired using swept-source optical coherence tomography system (PLEX Elite 9000; Carl Zeiss Meditec, Inc, Dublin, California, USA). **(A)** A horizontal line scans passing through the fovea was acquired in primary gaze. **(B)** Temporal and **(C)** nasal horizontal line scans were obtained in temporal and nasal gazes. Choroidal thicknesses were measured using a caliper tool at 1,000-μm intervals in the macular area and the peripheral areas in each direction. Irregular distribution of large choroidal vessels was observed frequently in the peripheral areas **(B,C)**.

Several efforts to evaluate CT in the peripheral retina have been made in addition to posterior pole and peripapillary area (113, 114). Development of faster OCT systems and wide-viewing technologies have enabled easier scanning of the far periphery (Figure 7) (31, 115–117). Mohler et al. (118) described choroidal images and CT maps in normal and diseased eyes over an ~60° field of view using prototype SS-OCT. However, this method using a special lens and device is difficult to apply in a general clinical environment. Using commercially available SD-OCT devices, Rasheed et al. (119) analyzed CT up to the mid-equator with manual montage and showed that the choroid outside the macula was significantly thinner in all quadrants than the choroid within the macula, with the greatest reduction observed in the inferior quadrant. Hoseini-Yazdi et al. (120) used a wide-field lens module and EDI-OCT to assess the CT throughout a 55 × 45° region centered on the fovea and found that the choroid thinned substantially toward the periphery, with minimum and maximum peripheral thinning superiorly and nasally, respectively. They also found that myopia-related choroidal thinning was more prominent in the macular than in the extra-macular areas. In addition to normal eyes, Singh et al. (29) conducted wide-field choroidal vessel analysis in CSC and fellow eyes and measured CT in extremes of gazes in all quadrants. They reported that the absolute value of CT was higher in CSC eyes than in normal eyes, however the topographical variation was similar to that of the normal eyes. In studies using SD-OCT, it is difficult to obtain multiple images at consecutive and parallel positions for manual montage, and image analysis requires substantial effort and time. Additionally, this method has the disadvantage of being difficult to acquire images from the same location in a longitudinal study.

Using SS-OCT, Breher et al. (121) generated a CT map of 13 extended wide-field ETDRS sectors with radii of 0.5, 1.5, 3, and 6 mm in myopic eyes and showed a topographic variation of CT with thicker choroid in the central, superior, and temporal regions. They also suggested that the elongation of the myopic eye influenced the CT. Kim et al. (30) used SS-OCT and a wide-field 16 mm line scan to measure the CT in the macular, nasal peripapillary, and peripheral areas. Although CT demonstrated bilateral symmetry in both the central and peripheral regions, the degree of CT asymmetry increased toward the nasal peripapillary and peripheral regions. Their results suggested that the nasal peripapillary and peripheral choroid had greater anatomic variation than the macula. In another study, Lim et al. (31) compared CT between the pachychoroid and normo-choroid groups using the same imaging protocol. They showed that the pachychoroid group had thicker choroid in both the macular and peripheral areas than the normo-choroid group, however the difference was smaller in the peripheral area. In their study, regional variations in the distribution of pachyvessels were observed more frequently in the nasal peripapillary and peripheral areas than in the macula (Figure 7). They speculated that these variations caused the topographic CT pattern of the nasal peripapillary and peripheral areas to differ from that of the subfovea, implying the fact that only the subfoveal CT could not represent the entire choroid of the eye. Wide-field OCT studies are still in their infancy, and more research on the characteristics and importance of the peripheral choroid is required. Peripheral distortion in OCT images is an important factor to consider when conducting and analyzing wide-field OCT research.

## Discussion

### Emerging Issues in Choroidal Thickness Measurements

The introduction of OCT has revolutionized the current practice of ophthalmology, and OCT imaging is now a standard method for investigating macular diseases. OCT devices using a faster and wider range of technologies have enabled the measurement of CT at various locations in the posterior eye. Similar to its contribution to the evaluation of macular thickness, wider and deeper choroidal imaging using the current OCT systems is now expected to open new avenues in CT measurement.

Most studies on CT measurement have focused on the measurement of subfoveal CT; however, CT measurement should not be limited to the macula for several reasons. First, most choroidal diseases are not confined to the macular area. Second, the anatomy and physiology of the choroid differ greatly from those of the retina (2). Although both the choroid and retina receive blood supply from the ophthalmic artery, the afferent and efferent vasculature are separated and located in different areas (1, 2, 122, 123). In the vascular system of the retina, the entry and exit points of vascular flow are located close to the optic nerve, whereas in the vascular system of the choroid, the entry and exit points are far apart. Moreover, the retinal vascular system has prominent avascular zones, such as the foveal avascular zone and peripheral avascular area, and many retinal vascular diseases are associated with flow defects in these areas. Meanwhile, the choroidal vascular bed has watershed zones situated between the various posterior ciliary arteries (PCA), short PCA, peripheral retrograde choroidal arteries, arterioles, and vortex veins (1, 122–124). Although the major posterior watershed zone of the choroid is usually situated between the nasal edge of the optic disc and the fovea in a vertical pattern (123, 125), it has been known to have considerable variations in location and configuration. Furthermore, a peripheral watershed zone between PCA and peripheral retrograde choroidal arteries has been identified in the temporal peripheral choroid near the equator (124). These various watershed zones, which can be found in various locations, from the macular to peripheral areas, have been associated with various types of chorioretinal disease (126–132). Despite these differences, subfoveal CT is a unique parameter representing the CT of an individual, similar to the central subfield thickness of the macula. Although one of the critical roles of the choroid is to supply blood to the retina in the foveal avascular zone and maintain the temperature of the macula (2), whether subfoveal CT is representative of the entire choroid of an individual still remains unclear. Third, another concern relates to the greater amplitude of physiological variation in subfoveal CT compared to that of the central subfield thickness of the macula (51, 133, 134), which is mainly derived from the diurnal change in the luminal area of the choroid (55). These findings have forced investigators to consider the time of the day when comparing subfoveal CT at different time points. In addition to diurnal variation, many factors, including age, sex, ethnicity, smoking, physical exertion, illumination, and alcohol consumption, can cause short-term changes in subfoveal CT (56–58, 135, 136). The finding that macular CT is vulnerable to physiological changes identifies the need to find a new way to calibrate CT measurements. Moreover, it remains questionable whether these variations are predictors of macular diseases or whether they are associated with them (137–139), though macular CT measurement has provided new insights into the understanding of macular diseases.

### Choroidal Thickness Profile

Choroidal blood flow changes dynamically in different physiological states (56–58, 136), and diurnal variations in CT are observed in the submacular choroid (50–56). Therefore, in order to characterize the choroid in specific conditions, it is necessary to take into account not only the primary factors influencing CT variation, but also the secondary factors as well. Moreover, rather than taking a single CT measurement at one point, taking multiple measurements at different locations which represent choroidal characteristics may provide more details about the choroid of a patient. Several efforts have been made to overcome the limitations of subfoveal CT measurements in isolation. In some studies, CT measurements at locations other than the macula or subfoveal CT have been attempted in various diseases (23, 79, 82, 89, 104, 108). In others, CT was measured both at the macula and outside the macular region (26, 32, 77, 81). These studies showed that variations in CT outside the macula can be different from those within it (81, 97–101). Another approach was to calculate the ratio of different CT measurements (32, 33, 75). Park et al. (140) used the CT ratio, defined as the ratio of peripapillary CT in a quadrant or clock-hour position to the average peripapillary CT of the individual, and they suggested that the CT ratio, rather than the absolute peripapillary CT value, could be more helpful in assessing regional peripapillary CT differences in eyes with normal tension glaucoma. Hwang et al. (141) proposed the choroidal spatial distribution index (CSDI) to characterize the topographic features or variations of the choroid. After measuring the choroidal volume in each subfield of the ETDRS grid, to account for the topographic variations, they calculated the vertical and horizontal CSDIs as the natural logarithm of the superior and temporal choroidal volumes divided by the inferior and nasal choroidal volumes, respectively. Yun et al. (26) suggested that the characteristics of nasal peripapillary CT outside the macula could differ from those of macular CT, since the blood flow of the nasal peripapillary differs from that of the macular area. Kim et al. (33) reported that the ratio of subfoveal to nasal CT was the best predictor of early response in the treatment of polypoidal choroidal vasculopathy. In another study (76), the authors showed that the ratio of subfoveal to nasal CT in eyes with chronic CSC was significantly lower than that in eyes with normal fundus, acute CSC, and resolved CSC.

Profiling is the term used to describe the activity of collecting important and useful details about someone or something, and profile is the term for a short description of someone's life, work, character, etc. Profiling chorioretinal diseases or systemic illnesses using a CT profile could prove useful for understanding the role of the choroid in the pathophysiology of these diseases. Several parameters for the CT profile have been suggested in previous studies. In addition to subfoveal CT, parafoveal CT has also been included in the CT profile (142–149). While macular CT varies with physiological state, CT outside the macula has been suggested to have a tendency to be less affected by macular diseases and treatments (31–33, 119). Regional differences in CT, such as the ratio of CT at the macula and outside the macula could be candidates for surrogates of the choroid (32, 33).

### Future Perspectives

The development of EDI-OCT and SS-OCT has ushered in a new era of *in vivo* and non-invasive CT measurements in humans. CT measurement is now a standard tool for characterizing the choroid in patients with ocular and systemic diseases. In addition to macular CT, CT outside the macula is expected to provide novel insights into the choroid. New parameters measured using OCT or OCT angiography, such as the choroidal vascularity index (150) and choriocapillaris flow void area (151), have recently been introduced for the characterization of the choroid, and these have helped us to better understand the choroid in normal and diseased eyes. Nevertheless, although more parameters can provide more information about the choroid, fewer parameters representing the choroid are easier to be adapted to busy daily clinics. In the future, we expect to determine which of the various choroidal parameters can more accurately and reliably represent the characteristics of the choroid in a particular disease. We also expect to determine which of these parameter combinations will help in the profiling of diseased eyes and patients.

## Author Contributions

JO designed the study and wrote the first draft of the manuscript. YK and JO searched and collected the data and contributed to manuscript revision. All authors read and approved final manuscript.

## Conflict of Interest

The authors declare that the research was conducted in the absence of any commercial or financial relationships that could be construed as a potential conflict of interest.

## Publisher's Note

All claims expressed in this article are solely those of the authors and do not necessarily represent those of their affiliated organizations, or those of the publisher, the editors and the reviewers. Any product that may be evaluated in this article, or claim that may be made by its manufacturer, is not guaranteed or endorsed by the publisher.
